# Identifying the Types of Ion Channel-Targeted Conotoxins by Incorporating New Properties of Residues into Pseudo Amino Acid Composition

**DOI:** 10.1155/2016/3981478

**Published:** 2016-08-18

**Authors:** Yun Wu, Yufei Zheng, Hua Tang

**Affiliations:** ^1^College of Computer and Information Engineering, Xiamen University of Technology, Xiamen 361024, China; ^2^Department of Pathophysiology, Southwest Medical University, Luzhou 646000, China

## Abstract

Conotoxins are a kind of neurotoxin which can specifically interact with potassium, sodium type, and calcium channels. They have become potential drug candidates to treat diseases such as chronic pain, epilepsy, and cardiovascular diseases. Thus, correctly identifying the types of ion channel-targeted conotoxins will provide important clue to understand their function and find potential drugs. Based on this consideration, we developed a new computational method to rapidly and accurately predict the types of ion-targeted conotoxins. Three kinds of new properties of residues were proposed to use in pseudo amino acid composition to formulate conotoxins samples. The support vector machine was utilized as classifier. A feature selection technique based on* F*-score was used to optimize features. Jackknife cross-validated results showed that the overall accuracy of 94.6% was achieved, which is higher than other published results, demonstrating that the proposed method is superior to published methods. Hence the current method may play a complementary role to other existing methods for recognizing the types of ion-target conotoxins.

## 1. Introduction

The marine cone snail can secrete venom for predation and defense. A key component of venom is called conotoxin which is a kind of disulfide-rich neurotoxic peptide with 10–30 residues long. The high diversity of their sequences makes it difficult to systemically study them. It has been reported that there are over 100,000 conotoxins existing in approximately 700 species of cone snails [1]. Conotoxins can target G protein-coupled receptors (GPCRs) [2], nicotinic acetylcholine, and neurotensin receptors. Particularly, they interact with ion channels with extremely high specificity and affinity [3]. Thus, they have been regarded as important drug candidates to treat chronic pain, epilepsy, spasticity, and cardiovascular diseases [4, 5].

With more and more conotoxins being discovered, biochemical experiments-based method to investigate the function of conotoxins becomes more and more difficult because of high cost and long period of wet experiment. Using computational method to predict the function of conotoxins provides us with a convenient way to perform systemic analysis of conotoxins. In 2006, Mondal et al. combined support vector machine (SVM) with pseudo amino acid composition (PseAAC) to predict the superfamily of conotoxins [6]. Subsequently, Lin and Li developed a novel method called increment of diversity (ID) to describe dipeptide sequence and used quadratic discriminant (QD) to predict superfamily and family of conotoxins [7]. Zaki et al. used sequence alignment which was also used by Zou et al. [8] combined with amino acid composition to predict superfamily of conotoxins by use of SVM [9]. They further provide a SVM-Freescore method to improve accuracy [10]. Recently, Yin et al. developed a method called dHKNN to predict superfamily of conotoxins and achieved the overall accuracy of 90.3% by using hidden Markov model to select best features [11, 12]. Lisacek et al. used profile Hidden Markov Models (pHMMs) and position-specific scoring matrix (PSSM) to improve accuracy for conotoxin superfamily prediction [13–15].

Although the methods and results mentioned above can give some guide to study conotoxins, they did not provide more information for the prediction of conotoxins' function. A case shows that two conotoxins (delta-conotoxin-like Ac6.1 and omega-conotoxin-like Ai6.2) belong to the same superfamily; however, they can target different ion channels [16]. Thus, it is necessary to develop new bioinformatics tools to identify the function of conotoxins. In 2007, Saha and Raghava proposed a method based on SVM and PSI-BLAST to predict the function of neurotoxins [17]. Soli et al. developed a statistical-based model to predict the activity of scorpion toxins by using motifs and secondary structure information [18]. Recently, Yuan et al. developed a feature selection technique based on binomial distribution to predict the types of ion channel-targeted conotoxins by using radial basis function network [19]. Subsequently, they improved the accuracy by using SVM with optimal dipeptide composition [20]. However, the prediction accuracy can be further improved.

Thus, the present study aimed to develop a new prediction method to improve the prediction quality of conotoxins' types. We incorporated three kinds of new properties of residues into PseAAC for formulating conotoxins samples. Subsequently, we used SVM to perform classification. After feature selection, we found that the accuracy was dramatically improved in jackknife cross-validation. In the following section, we will introduce the process of model construction in detail.

## 2. Materials and Methods

### 2.1. Benchmark Dataset

The benchmark dataset extracted from the UniProt [21] was constructed by Lin's group [19, 20]. The dataset is reliable and objective because (i) the conotoxins with ambiguous annotations have been excluded, (ii) the function of all conotoxins in benchmark dataset has been experimentally confirmed, and (iii) high similar sequences (cutoff = 80%) have been pruned by using CD-HIT program. The benchmark dataset contains 112 mature conotoxins peptide sequences including 24 potassium ion channel-targeted conotoxins (K-conotoxins), 43 sodium ion channel-targeted conotoxins (Na-conotoxins), and 45 calcium ion channel-targeted conotoxins (Ca-conotoxins). All calculations and model construction in the following section are based on the data.

### 2.2. Feature Extraction

A key point in protein prediction is how to extract important information from peptide sequences. In the past studies, the amino acid composition has been widely used in protein prediction. To consider the correlation of residues, the dipeptide composition was used in prediction model. Chou proposed a very popular and elegant descriptor called PseAAC which describes not only the correlation of physicochemical properties of residues but also the amino acid composition [22]. Furthermore, recently some web servers or stand-alone tools have been proposed to generate different modes of PseAAC, such as PseKNC [23], PseKNC-General [24], Pse-in-One [25], repRNA [26], and repDNA [27]. The authors should introduce these tools. In this study, we proposed three kinds of new properties, that is, rigidity, flexibility, and irreplaceability. The flexibility and rigidity of residues correlate with the protein structure and function. The irreplaceability of residues can reflect the evolution of life. The values of three properties for 20 residues [28] have been listed in Table 1. In the following, we will describe how to formulate conotoxins with PseAAC [22].

Consider a conotoxin **P** = *R*_1_ *R*_2_ *R*_3_ *R*_4_ ⋯ *R*_*L*_, where *R*_1_, *R*_2_, and *R*_*L*_ denote the 1st, 2nd, and *L*th residue of the conotoxin sample **P**; it can be defined by a 400 + 3*λ*-dimensional vector as shown by

(1) $$\begin{array}{c} P={\left[\;x_{1}\cdots x_{400}\cdots x_{400+3\lambda }\;\right]}^{T}, \end{array}$$

where

(2) xu=fu∑i=1400fu+w∑j=13λτj1≤u≤400ωτu∑i=1400fu+ω∑j=1nλτj400+1≤u≤400+3λ,

where *f*_*u*_ is the normalized frequency of the 400 dipeptides in conotoxin **P** and can be defined as

(3) $$\begin{array}{c} f_{u}=\frac{n_{u}}{\sum_{u}n_{u}}, \end{array}$$

where *n*_*u*_ denotes the number of occurrences of *u*th dipeptide in conotoxin **P**.

In (2), *ω* is weight factor for sequence order effect. *τ*_*j*_ is called the *j*-tier sequence correlation factor computed by the following formula:

(4) $$\begin{array}{c} \tau_{1}=\frac{1}{L-1}\overset{L-1}{\underset{i=1}{\sum }}H_{i,i+1}^{1},\\ \tau_{2}=\frac{1}{L-1}\overset{L-1}{\underset{i=1}{\sum }}H_{i,i+1}^{2},\\ \tau_{3}=\frac{1}{L-1}\overset{L-1}{\underset{i=1}{\sum }}H_{i,i+1}^{3},\\ \vdots \\ \tau_{3\lambda }=\frac{1}{L-\lambda }\overset{L-\lambda }{\underset{i=1}{\sum }}H_{i,i+\lambda }^{3}\\ \;\left(\lambda <L\right), \end{array}$$

where *H*_*i*,*i*+*λ*_^*n*^  (*n* = 1,2, 3 denotes rigidity, flexibility, and irreplaceability) is called the correlation function and can be given by

(5) $$\begin{array}{c} H_{i,i+\lambda }^{n}=h^{n}\left(\;R_{i}\;\right)\cdot h^{n}\left(\;R_{i+\lambda }\;\right), \end{array}$$

where *h* ^*n*^(*R*_*i*_) is the *n*th kind of the physicochemical values of the amino acid *R*_*i*_. The values should be converted to standard type by

(6) $$\begin{array}{c} h^{n}\left(\;R_{i}\;\right)⟸\frac{h_{0}^{n}\left(R_{i}\right)-\langle h_{0}^{n}\left(R_{i}\right)\rangle }{SD\langle h_{0}^{n}\left(R_{i}\right)\rangle }, \end{array}$$

where *h*_0_^*n*^(*R*_*i*_) is the original physicochemical values of the *i*th amino acid.

For the purpose of finding the best feature subset which can produce the maximum accuracy, we performed feature selection by using the algorithm called *F*-score which can be defined as

(7) $$\begin{array}{c} F\left(\;i\;\right)=\frac{\sum_{k=1}^{3}{\left({\overset{-}{x}}_{i}^{k}-{\overset{-}{x}}_{i}\right)}^{2}}{\sum_{k=1}^{3}\left(1/\left(N_{k}-1\right)\right)\sum_{j=1}^{N_{k}}{\left(x_{ij}^{k}-{\overset{-}{x}}_{i}^{k}\right)}^{2}}, \end{array}$$

where ${\overset{-}{x}}_{i}$ and ${\overset{-}{x}}_{i}^{k}$ are the average values of the *i*th feature in whole dataset and the *k*th dataset; *x*_*ij*_^*k*^ is the value of the *i*th feature of the *j*th conotoxin in the *k*th dataset; and *N*_*k*_ is the numbers of conotoxin in the *k*th dataset. We noticed that the larger the *F*(*i*) value is, the better the predictive capability the *i*th feature has. We used a python script fselect.py downloaded from https://www.csie.ntu.edu.tw/~cjlin/libsvmtools/ to calculate *F*-score.

### 2.3. Support Vector Machine

SVM is a very popular machine learning method which is very suitable for small sample classification [29–31] and regressions [32, 33]. Its basic idea is to map the original samples into a high-dimensional space and search for the best hyperplane in this space which can separate different samples. In this study, the LibSVM soft package was used to implement SVM. The radial basis function (RBF) usually exhibits excellent performance in nonlinear classification [34]. Thus the RBF kernel function was used in the current work. We utilized grid search method to find out the best values of the regularization parameter *C* and kernel parameter *γ* via jackknife cross-validation. The search spaces for *C* and *γ* are [2^15^, 2^−5^] and [2^−5^, 2^−15^] with steps being 2^−1^ and 2, respectively.

### 2.4. The Evaluation of Model Performance

We used jackknife cross-validation to evaluate the performance of proposed method. Three metrics, namely, sensitivity (Sn), overall accuracy (OA), and average accuracy (AA) as defined in [19, 20], were used to quantitatively estimate the accuracy of the model:

(8) Snk=mkNk,OA=∑k=13mk∑k=13Nk,AA=Snk3,

where *N*_*k*_ is the total number of the *k*th types of conotoxins and *m*_*k*_ denotes the number of the *k*th types of conotoxins which was correctly recognized.

## 3. Results and Discussion

As we can see from (2), the results of the proposed method depend on two parameters *λ* and *ω*, where *λ* represents the long-range sequence order effect and *ω* is called weight factor which reflects the weight imposed between the local and global effects. Generally speaking, the greater *λ* is, the more global sequence order information it contains. However, if *λ* is too large, it would cause the high-dimensional disaster as mentioned above. Therefore, our searching for the optimal values of the three parameters was carried out in the following regions:

(9) 1≤λ≤10with  step  Δ=10.1≤w≤1.0with  step  Δ=0.1.

From (9), a total of 10 × 10 = 100 individual combinations needed to be considered for finding the optimal parameter combination. This was actually a routine but tedious process to optimize the model via a 2-dimensional grid search. We used the jackknife cross-validation approach to deal with the parameter optimization. The results show that when *λ* = 6 and *ω* = 0.2, the accuracy reaches to maximum value. We noticed that the current model contains 418 features which is still so large that the high-dimensional and overfitting problems will appear.

Therefore, we must select the key features from the 418 components. These key features can produce the maximum Acc. The best feature subset will be obtained by investigating all the combinations of features. However, it is time-consuming and even beyond computational capability for most computers to examine all possible combinations. Based on this reason, we used *F*-score defined in (7) to perform feature selection. At first, all 418 features were ranked according to their *F*-scores from large to small. Secondly, the SVM was used to classify three samples and calculate the accuracy based on the feature with maximum *F*-score. Thirdly, a new feature subset was produced by adding the feature with the second highest* F* value to the former feature subset. We repeated the process until all combinations were investigated and the accuracies were calculated.

**Figure 1:**
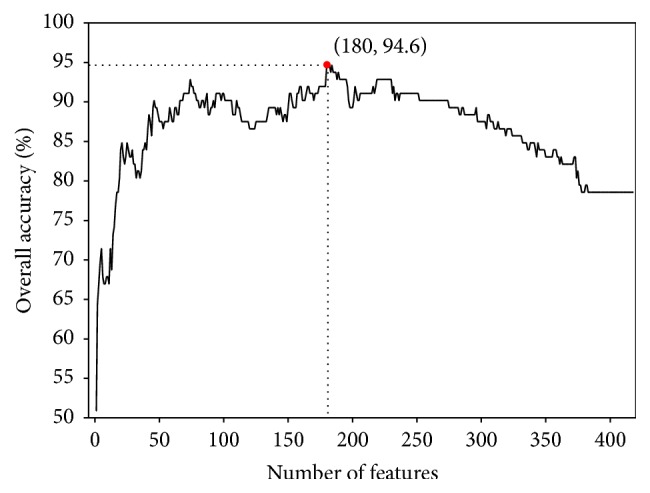
A plot to show the feature selection results. When the top 180 features were used to perform prediction, the overall success rate reached its peak of 94.6%.

We plotted the accuracies with feature dimension in Figure 1 and noticed that the maximum accuracy is 94.6% when 180 best features were used. The detailed results were recorded in Table 1. Other published results were also listed in Table 2. We noticed that Sns of Na- and Ca-conotoxins of our method are 95.3% and 95.6%, respectively, which are higher than those of RBF network-based method [19]. The Sns of K- and Ca-conotoxins of our method are 91.7% and 95.6%, respectively, which are higher than those of iCTX-Type [20]. Thus, in summary, our proposed method is superior to other published methods.

## 4. Conclusion

In this paper, we designed a new method based on three kinds of new properties to predict three kinds of ion channel-targeted conotoxins. By using feature selection technique, prediction accuracy was dramatically improved. Comparison with published methods demonstrated the advantage of our method. The properties of residues used in this paper can also be used in other fields of protein classification. In the future, we will construct a free webserver based on the proposed method for the convenience of the vast majority of experimental scientists.

## Figures and Tables

**Table 1:** The values of rigidity, flexibility, and irreplaceability of 20 residues.

Residues	Rigidity	Flexibility	Irreplaceability
G	−1.097	−2.746	0.56
A	−1.338	−3.102	0.52
V	−1.641	−1.339	0.54
L	−1.741	0.424	0.58
I	−1.741	0.424	0.65
F	2.877	−0.466	0.86
W	5.913	−1.000	1.82
Y	2.714	−0.672	0.98
D	−0.204	0.424	0.77
H	2.269	−0.223	0.94
N	−0.204	0.424	0.79
E	−0.365	2.009	0.76
K	−1.822	3.950	0.81
Q	−0.365	2.009	0.86
M	−1.741	2.484	1.25
R	1.169	3.06	0.6
S	−1.511	0.957	0.64
T	−1.641	−1.339	0.56
C	−1.511	0.957	1.12
P	1.979	−2.404	0.61

**Table 2:** Comparison of the current method with published methods.

Methods	Sn (%)			AA (%)	OA (%)
	K	Na	Ca		
RBF network [19]	91.7	88.3	88.9	89.7	89.3
iCTX-Type [20]	83.3	97.8	89.8	90.31	91.1
Our method	91.7	95.3	95.6	94.2	94.6
